# Standard metadata for 3D microscopy

**DOI:** 10.1038/s41597-022-01562-5

**Published:** 2022-07-27

**Authors:** Alexander J. Ropelewski, Megan A. Rizzo, Jason R. Swedlow, Jan Huisken, Pavel Osten, Neda Khanjani, Kurt Weiss, Vesselina Bakalov, Michelle Engle, Lauren Gridley, Michelle Krzyzanowski, Tom Madden, Deborah Maiese, Meisha Mandal, Justin Waterfield, David Williams, Carol M. Hamilton, Wayne Huggins

**Affiliations:** 1 Biomedical Applications Group, Pittsburgh Supercomputing Center, 300 S Craig Street, Pittsburgh, PA 15213 USA; 2 Department of Physiology, University of Maryland School of Medicine, 660 West Redwood Street, Baltimore, MD 21201 USA; 3 Centre for Gene Regulation & Expression, Division of Computational Biology, University of Dundee, Nethergate, Dundee, Scotland DD1 4HN United Kingdom; 4 Morgridge Institute for Research, 330 N Orchard Street, Madison, WI 53715 USA; 5 Cold Spring Harbor Laboratory, One Bungtown Road, Cold Spring Harbor, NY 11724 USA; 6 Mark and Mary Stevens Neuroimaging and Informatics Institute, Laboratory of Neuro Imaging, Keck School of Medicine of University of Southern California, 1975 Zonal Avenue, Los Angeles, CA 90033 USA; 7 Bioinformatics and Computational Biology Program, RTI International, 3040 East Cornwallis Road, Research Triangle Park, NC 27709 USA

**Keywords:** Neuroscience, Standards

## Abstract

Recent advances in fluorescence microscopy techniques and tissue clearing, labeling, and staining provide unprecedented opportunities to investigate brain structure and function. These experiments’ images make it possible to catalog brain cell types and define their location, morphology, and connectivity in a native context, leading to a better understanding of normal development and disease etiology. Consistent annotation of metadata is needed to provide the context necessary to understand, reuse, and integrate these data. This report describes an effort to establish metadata standards for three-dimensional (3D) microscopy datasets for use by the Brain Research through Advancing Innovative Neurotechnologies® (BRAIN) Initiative and the neuroscience research community. These standards were built on existing efforts and developed with input from the brain microscopy community to promote adoption. The resulting 3D Microscopy Metadata Standards (3D-MMS) includes 91 fields organized into seven categories: Contributors, Funders, Publication, Instrument, Dataset, Specimen, and Image. Adoption of these metadata standards will ensure that investigators receive credit for their work, promote data reuse, facilitate downstream analysis of shared data, and encourage collaboration.

## Introduction

New fluorescence microscopy techniques, coupled with recent advances in tissue clearing, labeling, and staining provide unprecedented opportunities to investigate brain structure and function. These technologies have been used generate high-resolution, three-dimensional (3D) images of entire brains from model organisms^1–8^ and large sections of brains from humans^9,10^. Data from these images have been used for cell-type-specific mapping of the brain connectome^11^, identifying and characterizing brain regions governing behavior^12–14^, and defining structural aspects of disease pathologies^9^. The number of 3D microscopy datasets will grow exponentially with the introduction of major brain initiatives from countries around the world. The results of these efforts will make it possible to catalog brain cell types and define their location, morphology, and connectivity in a native context, leading to a better understanding of normal development and disease etiology.

To maximize the utility of these data, it will be essential to establish and adopt standards for annotating, reporting, and formatting imaging datasets. Such standards can facilitate scientific transparency, rigor, and reproducibility; aid data sharing and integration; and promote the development of analysis tools and a sustainable informatics ecosystem. There are several microscopy standards efforts currently underway. Example community standards include minimum information guidelines for fluorescence microscopy developed within the 4D Nucleome (4DN) project^15^, minimal metadata and data structures developed within the National Institutes of Health (NIH) Common Fund’s Stimulating Peripheral Activity to Relieve Conditions (SPARC) project^16^, data structures for two-dimensional (2D) and 3D microscopy being developed under the Brain Imaging Data Structure Extension Proposal 031, and metadata guidelines from Recommended Metadata for Biological Images^17^. Other relevant efforts include QUality Assessment and REProducibility for instruments and images in Light Microscopy (QUAREP-LiMi), which aims to improve the overall quality and reproducibility of microscopy datasets;^18,19^ the Global BioImaging initiative, which is establishing recommendations and guidelines for image data repositories and formats^20^; and the International Neuroinformatics Coordinating Facility (INCF), which coordinates neuroinformatics infrastructure and standards^21^.

This article describes an effort to establish metadata standards for 3D microscopy datasets for use by the Brain Research through Advancing Innovative Neurotechnologies® (BRAIN) Initiative and larger neuroscience research community. The metadata standards were developed by a 10-member Working Group (WG; https://doryworkspace.org/WorkingGroupRoster) with diverse expertise using a consensus-based process that relied on input from the scientific community. The metadata standards build on existing efforts, including the DataCite metadata schema (https://schema.datacite.org/) and the Open Microscopy Environment (OME)^22^, to promote adoption and utility. The resulting 3D Microscopy Metadata Standards (3D-MMS) is designed to ensure that a 3D microscopy dataset is sufficiently described to support its reuse by scientists who did not generate the data. These metadata standards are being implemented within the Brain Image Library (BIL)^23^, the designated repository to accept and make microscopy data publicly available for investigators funded by the BRAIN Initiative. Adoption of these metadata standards will aid investigators who want to share data, helping them to evaluate and decide which data can be combined. The use of these metadata standards also ensures that datasets comply with FAIR (**F**indable, **A**ccessible, **I**nteroperable, and **R**eusable) principles^24^ and the BRAIN Initiative data sharing policy^25^.

## Results

3D-MMS includes 91 metadata fields organized into seven categories: Contributors, Funders, Dataset, Image, Instrument, Publication, and Specimen. Each metadata field is specified by a name, a definition, a list of allowable values, whether it is required or optional for submission to the BIL (https://www.brainimagelibrary.org), and the number of times it can be repeated for a dataset. The complete metadata specification is available from the Defining Our Research Methodology (DORy) website (https://doryworkspace.org/metadata).

To encourage adoption and reduce burden on data submitters, only 31 of the fields in 3D-MMS are required for submission of a dataset to the BIL. Nineteen of the required metadata fields support assignment of a Digital Object Identifier (DOI) to the dataset. A DOI is a unique code that provides a permanent and stable mechanism for retrieval of a dataset and its metadata^26^. The remaining required fields are necessary for investigators to open and view the images and to determine if they want to reuse the dataset. A summary of the required metadata fields in each category is presented in Table 1.

**Table 1:** 3D Microscopy Metadata Standards (3D-MMS) Required Fields.

Field Name	Definition	Allowable Values	Supports Digital Object Identifier (DOI)?
**Contributors Category (nine required metadata fields)**			
contributorName	Person (last name, first name) or organization (e.g., research group, department, institution) contributing to or responsible for the project, but does not include funders of the project. If a contributor has more than one contributorType, use a separate line for each.	Free text	Yes
Creator	Main researchers involved in producing the data. There must be at least one creator.	Yes; No	Yes
contributorType	Categorization of the role of the contributor. Recommended: ProjectLeader (for principal investigator), ResearchGroup (for laboratory, department, or division).	ContactPerson; DataCollector; DataCurator; ProjectLeader; ProjectManager; ProjectMember; RelatedPerson; Researcher; ResearchGroup; Other	Yes
nameType	Type of contributorName.	Organizational; Personal	Yes
nameIdentifier	Alphanumeric code that uniquely identifies an individual or legal entity, (listed in the contributorName field). Accepted identifiers include GRID*, ISNI*, ORCID*, ROR*, and RRID*. Preferred identifiers are ORCID for personal names and ROR for organizational names. Required for Personal nameType.	Free Text	Yes
nameIdentifier Scheme	Identifying scheme used in nameIdentifier. Required for Personal nameType.	GRID*; ISNI*; ORCID*; ROR*; RRID*	Yes
affiliation	Organizational or institutional affiliation of the contributor.	Free text	Yes
affiliationIdentifier	Unique identifier (ROR preferred) for the organizational or institutional affiliation of the contributor.	Free text	Yes
affiliationIdentifierScheme	Identifying scheme used in affiliationIdentifier.	GRID*; ISNI*; ORCID*; ROR*; RRID*	Yes
**Dataset Category (five required metadata fields)**			
Title	Short phrase by which the specific dataset is known (e.g., title of a book).	Free text	Yes
Rights	Any rights information for the dataset. May be the name of the license and can include embargo or other use restrictions on data (see https://spdx.org/licenses)	Free text	Yes
rightsURI	If using a common license and licensing information is online, provide a link to the license.	Free text	Yes
rightsIdentifier	If using a common license, provide the Software Package Data Exchange (SPDX) code for the license (see https://spdx.org/licenses).	Free text	Yes
Abstract	Additional descriptive information about the dataset, including a brief description and the context in which it was created (e.g., aim of the experiment, what the dataset is expected to show). This abstract will be used on the DOI landing page and will be the primary description of the dataset; it will ideally be more than 100 words.	Free text	Yes
**Funders Category (five required metadata fields)**			
funderName	The name of the funder.	Free text	Yes
fundingReferenceIdentifier	Alphanumeric code that uniquely identifies an individual or legal entity. Preferred identifier is ROR.	Free text (or URL)	Yes
fundingReferenceIdentifierType	Identifying scheme used in fundingReferenceIdentifier.	GRID*; ISNI*; ORCID*; ROR*; RRID*	Yes
awardNumber	Funding code or project number assigned to the grant.	Free text	Yes
awardTitle	Title of the grant award.	Free text	Yes
**Instrument Category (two required metadata fields)**			
MicroscopeType	Type of microscope used to capture the image (e.g., inverted, upright, light sheet, confocal, two photon).	Free text	No
MicroscopeManufacturerAndModel	Manufacturer and model of the microscope used.	Free text	No
**Image Category (eight required metadata fields)**			
xAxis	Predominant tissue direction as one moves from the left side of the image to the right side of the image.	Left to right; Right to left; Anterior to posterior; Posterior to anterior; Inferior to superior; Superior to inferior; Oblique	No
yAxis	Predominant tissue direction as one moves from the top of the image to the bottom of the image.	Left to right; Right to left; Anterior to posterior; Posterior to anterior; Inferior to superior; Superior to inferior; Oblique	No
zAxis	Predominant tissue direction as one follows a given pixel position through the stack of images from the first image to the last image.	Left to right; Right to left; Anterior to posterior; Posterior to anterior; Inferior to superior; Superior to inferior; Oblique	No
Number	Number assigned to each channel.	Free text	No
displayColor	Original display color for rendering each channel in triplet (red, green, blue) format.	Free text	No
stepSizeX	Physical step size in X dimension (e.g., pixel size represents how many microns).	Free text	No
stepSizeY	Physical step size in Y dimension (e.g., pixel size represents how many microns).	Free text	No
stepSizeZ	Distance between the center of one image and the center of adjacent images in Z dimension (space in microns between slices).	Free text	No
**Specimen Category (five required metadata fields)**			
Species	Common organism classification name for the donor organism (e.g., mouse, human).	Free text	No
NCBITaxonomy	National Center for Biotechnology Information (NCBI) taxonomy code for species of the donor organism.	Free text	No
Age	Age of the donor (or unknown).	Number	No
Ageunit	Unit for the age of the donor.	Days; Months; Years	No
Sex	Sex of the donor.	Male; Female; Unknown	No

*For more information on the identifiers, see the Global Research Identifier Database (GRID, https://www.grid.ac); International Standard Name Identifier (ISNI, https://isni.org); Open Researcher and Contributor ID (ORCID, https://orcid.org); Research Organization Registry (ROR, https://ror.org); and Research Resource Identifiers (RRID, https://scicrunch.org/resources) websites.

### Contributors

The Contributors category includes nine required metadata fields that identify the scientists and organizations involved in the creation of the dataset (https://doryworkspace.org/metadata, Table 1). Contributors include the broader set of researchers and institutions (except funders) that participated in the development of the dataset. Each contributor is identified by name; role on the project (e.g., data collection, management, and/or distribution); and affiliation. The Creator field is used to indicate whether a contributor is also a creator (“Yes” or “No”). Creators are the principle researchers involved in the generation of the dataset. There must be a least one creator for each dataset. All nine metadata fields in the Contributors category, including controlled vocabulary and references to digital identifiers (e.g., Open Researcher and Contributor ID [ORCID], Research Resource Identifiers [RRID]), are equivalent to properties in the DataCite metadata schema (https://schema.datacite.org/) and support assignment of a DOI to the dataset.

### Funders

The Funders category includes five metadata fields that describe the organizations providing financial support for the generation of the dataset (https://doryworkspace.org/metadata, Table 1). The five Funders metadata fields are only required if the project is funded by government agencies, such as the NIH. The WG recognized that some information (e.g., award number) may not be available for projects funded by foundations and internal mechanisms. All the Funders metadata fields, including those uniquely identifying organizations (e.g., ORCID, RRID), are equivalent to properties in the DataCite metadata schema (https://schema.datacite.org/) and support assignment of a DOI to the dataset.

### Dataset

The Dataset category includes 15 metadata fields that provide a high-level description of the data (e.g., title, abstract, methods, imaging modality) and how the data can be reused (https://doryworkspace.org/metadata). The WG defines a dataset as a collection of images (in one or more files) generated from a light microscope that can be stacked to form a distinct 3D volume or object, such as a brain region or an entire brain. Relevant 3D light microscope technologies include (but are not limited to) scanning confocal microscopy (point or line scanning), spinning disk confocal microscopy, multiphoton excitation microscopy (2p or 3p), light sheet microscopy (selective/single plane, oblique, ultramicroscopy), structured illumination, and deconvolved widefield. Five of the Dataset metadata fields are required, including title, abstract, and those describing the intellectual property rights (Table 1). Optional items include fields to describe modality (e.g., morphology, connectivity), technique (e.g., anterograde tracing, single molecule fluorescence *in situ* hybridization), and methods used to generate biological materials and computationally process the data. Eight of the metadata fields in the Dataset category are equivalent to properties in the DataCite metadata schema (https://schema.datacite.org/) and support assignment of a DOI to the dataset.

### Image

The Image category includes 33 metadata fields that describe the size and 3D orientation of the image, channels and fluorophores used, and location of relevant landmarks (https://doryworkspace.org/metadata). Eight of the Image metadata fields are required, including those describing the x, y, and z orientation, channel numbers, colors, the number of microns per pixel in the x and y dimensions, and the space in microns between slices in the z dimension (Table 1). Optional fields include items to describe oblique dimensions (if applicable), the name and coordinates of landmarks, the number of pixels in the x, y, and z dimension, and the number of files, timepoints, channels, and slices. Fifteen of the metadata fields in the Image category are equivalent to properties in the OME Data Model^22^.

### Instrument

The Instrument category includes 12 metadata fields that describe the instrument used to capture the images (https://doryworkspace.org/metadata). There are two required fields to record the microscope type and model (Table 1). Optional fields record the details of the objective, detector, type of illumination and wavelength, and temperature of the sample. All fields in the instrument category take free text, but there are suggested values for describing the objective immersion medium, type of illumination, and type of detector. All the Instrument metadata fields are equivalent to properties in the OME Data Model^22^.

### Publication

The Publication category includes five optional metadata fields that identify publications, preprints, and protocols that are related to the dataset (https://doryworkspace.org/metadata). Each publication, preprint, or protocol can be identified by a globally unique identifier (e.g., DOI, International Standard Book Number [ISBN]), a PubMed Central identifier (if applicable), and/or a citation. Three of the Publication metadata fields, including those describing the unique identifier and the relationship of the publication, preprint, or protocol to the dataset, are equivalent to properties in the DataCite metadata schema (https://schema.datacite.org/) and support assignment of a DOI to the dataset.

### Specimen

The Specimen category includes 12 metadata fields that describe the donor, organ, and sample being studied (https://doryworkspace.org/metadata). Five of the Specimen metadata fields are required, including those to record the common name, National Center for Biotechnology Information taxonomy code, age, and sex of the organism being studied (Table 1). Optional fields include items to capture the genotype of the organism, organ name, location or region where the sample is found, and name of the atlas used to describe the location (if applicable).

#### JavaScript object notation (JSON) specification for 3D-MMS

A formal specification for 3D-MMS in JSON Schema is available to the research community on GitHub (https://github.com/Defining-Our-Research-Methodology-DORy/3D-Microscopy-Metadata-Standards-3D-MMS). The GitHub repository includes a separate JSON Schema for each of the seven metadata categories as well as an overall JSON Schema that incorporates all categories (record_schema.json). For each JSON Schema, there is a corresponding file with example JSON-formatted metadata. The GitHub repository includes python validation scripts that compare a user’s JSON-formatted metadata files against the schemas and returns details of any errors. Users can also record metadata utilizing the Excel template on the GitHub repository and convert to JSON format using the accompanying python script.

#### Implementation of 3D-MMS in the brain image library

BIL is an NIH-funded national public resource enabling researchers to deposit, analyze, mine, share, and interact with large brain image datasets^23^. BIL is the designated repository to accept and make microscopy data publicly available for investigators funded by the BRAIN Initiative. BIL’s process for collecting metadata uses a submission portal that collects required metadata through a combination of values submitted directly in the portal and a multi-tabbed metadata spreadsheet that is uploaded through a submission portal and linked to submitted data. The existence of BIL predates this standard; thus, the existing metadata collected within BIL is only a small subset of the metadata described here.

BIL first piloted the collection of metadata aligned with 3D-MMS among a subset of existing data contributors. Within this pilot, investigators were asked to review and expand a multi-tabbed metadata spreadsheet that was prepopulated with information from their submitted datasets along with the existing BIL metadata mapped onto the new metadata schema. From the pilot, it was found that the data contributors could provide the minimally requested information, but there were areas within the collection spreadsheet where the instructions were unclear and misinterpreted.

Additional feedback received led to the implementation of a series of “suggested values” for free-text fields to help better categorize the data being received by the archive. Examples of suggested values are the imaging modality names (https://github.com/BICCN/BCDC-Metadata/blob/master/Data-Collection-Inventory/2021Q3_csvs/modality.csv) and imaging technique names (https://github.com/BICCN/BCDC-Metadata/blob/master/Data-Collection-Inventory/2021Q3_csvs/technique.csv) used by the BRAIN Initiative Cell Census Network (BICCN) project.

**Fig. 1:**
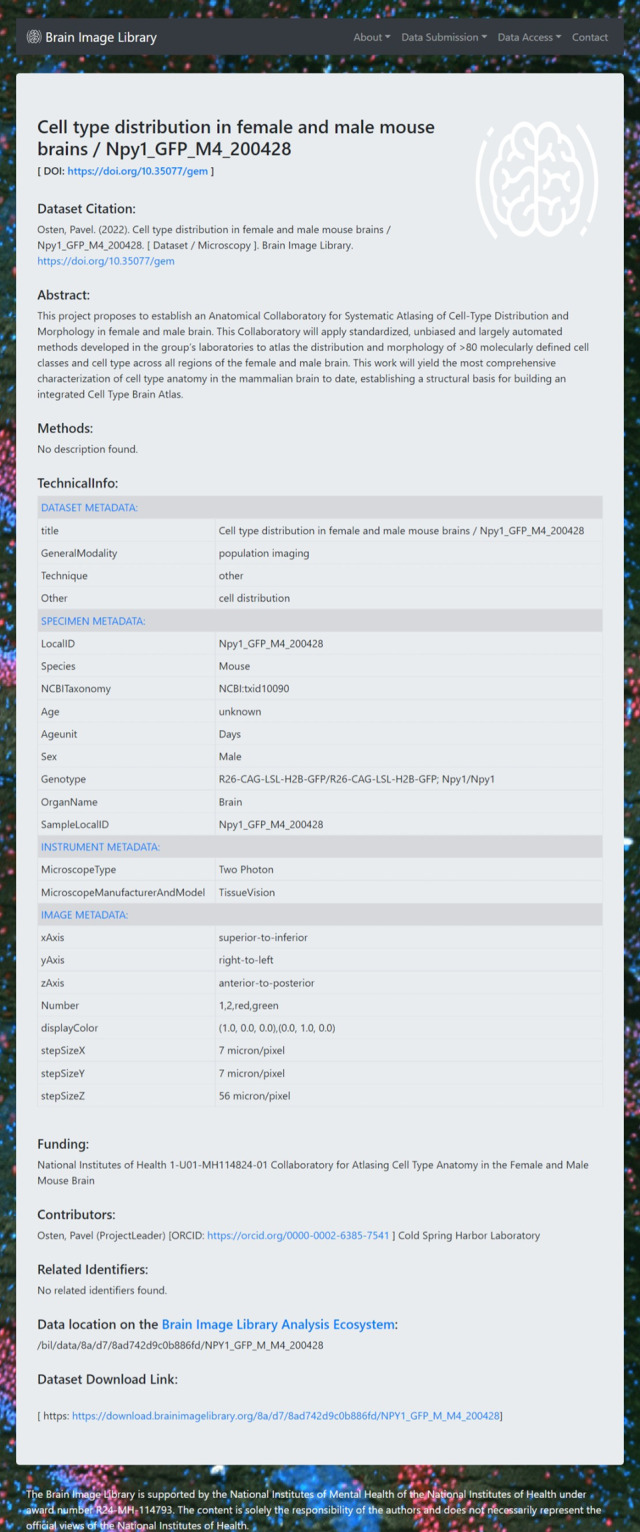
A Brain Image Library dataset annotated with 3D-MMS.

BIL fully implemented the ingestion process and began requiring the use of 3D-MMS for all new data submissions in early 2022. Figure 1 shows an example BIL dataset annotated with 3D-MMS. As of the date of this writing, 53 datasets have been made publicly available that use 3D-MMS. In addition to new datasets, BIL has begun contacting existing data contributors to collect additional metadata where possible that will align metadata from legacy datasets as close as possible to 3D-MMS.

## Discussion

Adoption of the 3D-MMS will provide several benefits to the BRAIN Initiative and larger neuroscience community. Consistent implementation of these standards will help ensure that 3D microscopy datasets are open, accessible, and sufficiently described to support reuse. Datasets annotated with rich metadata are a key component of FAIR principles and help users interpret an experiment and determine whether datasets can be combined for integrative secondary analyses. Twenty-seven of the 91 metadata fields, including 19 of the 31 required fields (Table 1), are equivalent to properties in the DataCite metadata schema (https://schema.datacite.org/) and support assignment of a DOI to a dataset. Most commonly associated with journal articles, DOIs provide a generic infrastructure for identifying any content over digital networks^26^. Because DOIs are persistent identifiers, users can reliably locate, share, and cite datasets even if URLs within a repository change. DOIs and associated metadata are also indexed in common search engines like Google. The freely available Crossref Event Data service tracks citation of dataset DOIs in publications and on scholarly websites^27^. These usage data can help data generators, repositories, and funders measure the use and impact of datasets over time. Repositories can add subsequent citations to datasets using the metadata fields in the Publications category to provide users with additional context. Adoption of the 3D-MMS can also help ensure that datasets are interoperable with other high-priority microscopy projects and efforts. Forty-one of the 91 metadata fields within the Essential Metadata for 3D BRAIN Microscopy are either equivalent, or can be mapped, to terms in the OME Data Model^22^. OME is a well-established informatics framework for storing and sharing biological microscopy data that is widely used in the United States and international research communities. The OME Data Model is being used in the Bioimaging in North America Network (https://www.bioimagingna.org) and several related large-scale microscopy projects (e.g., SPARC^16^ and the 4DN project^15^).

Next steps will focus on promoting adoption of 3D-MMS, improving metadata submission to the BIL, and evaluating gaps to be addressed in future versions of the standard. In addition to standard dissemination methods (e.g., publications, conferences, and talks by WG members), 3D-MMS has been submitted to the International Neuroinformatics Coordinating Facility (INCF) for review and endorsement. INCF review includes evaluation of the standard against the FAIR criteria, a community feedback step, and suggestions for improvement where needed. INCF promotes adoption of endorsed standards to the international neuroscience community, funders, and journals through organized outreach and training activities.

Within the BIL, plans include reducing the data entry burden and improving consistency of collected metadata. This includes collecting Contributors and Funders metadata within the BIL submission portal itself and creating a mechanism to attach this metadata to a series of datasets. This improvement would help groups, such as the BICCN, that have investigators depositing new entries over a period of months or years. There is also the possibility to reduce data entry further by building lookup capabilities into the portal that might, for example, query external portals that have restful application programming interfaces (e.g., the NIH Research Portfolio Online Reporting Tool Expenditures and Results [RePORTER], https://projectreporter.nih.gov/) or project-specific resources on GitHub.

3D-MMS will be periodically updated to ensure that it continues to meet the needs of the scientific community. Potential updates include capturing additional experimental details (e.g., data processing and analysis), addressing emerging technologies, and incorporating suggestions from the user community (e.g., community feedback through the DORy website or the GitHub page). Future versions of the standards will be developed in collaboration with the scientific community. This could include a future iteration of the 3D Brain Microscopy WG, targeted conference workshops, or crowdsourcing efforts. Updates will be tracked according to the three-digit Major.Minor.Patch Semantic Versioning model. Major version changes (i.e., updates that are not compatible with previous versions) will be shared with the community for feedback before being finalized. Minor version changes (i.e., compatible with previous versions) may also be shared with the community for feedback depending on the nature of the updates. Patch releases will address errors and inconsistencies and will be handled by the project team. All updates will be documented in descriptive release notes on DORy and GitHub.

3D-MMS will help ensure that microscopy data from the BRAIN Initiative and larger neuroscience community are consistently annotated and aligned with FAIR principles. This will maximize the utility of these data and facilitate integrative analyses to understand the structure and functioning of the brain.

## Methods

3D-MMS was developed by the BRAIN Initiative 3D Microscopy WG between October 2019 and August 2020. The WG consisted of 10 members from the BICCN and the larger scientific community. WG members’ expertise included informatics, 3D microscopy, neuroscience, physiology, and engineering and spanned the experimental lifecycle (e.g., data generation, data archiving, data integration, and data analysis). This expertise helped ensure that the WG represented the BRAIN Initiative and the larger neuroscience research community.

The WG held an initial in-person meeting in October 2019 to define the scope of the standard and to establish the framework for prioritizing metadata attributes. During this meeting, the WG discussed the needs and perspectives of the user community and heard presentations on the BRAIN Initiative, BICCN, BIL, and OME. The WG discussed several key factors to be considered in the development of the 3D-MMS. First, the standards should support the goals of the BRAIN Initiative and the larger neuroscience research community. Second, the standards should build on existing standards efforts. Third, a subset of metadata should be required for all imaging datasets. Finally, the standards should be flexible enough to address the diversity of microscopy techniques within the BICCN and larger research community, including those not yet invented.

Following the in-person meeting, a metadata subgroup was established to develop an initial set of metadata fields and attributes. Between November 2019 and January 2020, the subgroup reviewed experimental modalities and technologies and specified use cases for querying, understanding, and reusing microscopy datasets. The subgroup also reviewed related metadata standardization efforts, including the OME^22^, Neuroimaging Data Model^28^, Brain Imaging Data Structure^29^, Force11^30^, and DataCite (https://schema.datacite.org/). Relevant metadata fields were captured in a spreadsheet that included the name of the metadata field, category, subcategory (if applicable), occurrence (whether the metadata field is required and how many times it can appear for each dataset), whether the metadata field is needed to generate a DOI, definition, units (if applicable), and allowable values (e.g., free text or controlled vocabulary lists).

The metadata subgroup presented its initial recommendations to the WG during an in-person meeting in February 2020. The WG provided feedback on the proposed metadata fields and attributes, including which fields should be required and whether any should be added, removed, or clarified. The WG also recommended that future efforts include additional metadata fields to address reproducibility and quality control. At the end of the meeting, the WG approved the preliminary draft of the standard.

The preliminary metadata standards were posted on the DORy website for feedback and comment by the larger research community between April 21 and May 12, 2020. Potential reviewers were identified by searching the NIH RePORTER (https://projectreporter.nih.gov/) and the BRAIN Initiative website (https://braininitiative.nih.gov/) for funded projects that included keywords for relevant microscopy techniques. These investigators were sent an email describing the project and asking them to provide feedback. Respondents were asked to review the metadata fields and attributes and complete Likert-style scales to indicate whether the proposed standards were sufficient and easy to adopt and implement. Respondents could also provide general comments and suggestions.

The WG reviewed outreach results during a teleconference in May 2020. A total of 42 individuals provided comments on the preliminary standard. In general, the response to outreach was positive, and numerous helpful comments and suggestions were offered. Between June and August 2020, the metadata subgroup met several times to review community input and make updates before finalizing version 1.0 of the 3D-MMS.

## Data Availability

Brain Image Library datasets are available at https://www.brainimagelibrary.org/.

## Code availability

All custom software, including the python code to transfer csv-formatted data to JSON and to validate JSON formatted data, are freely available at https://github.com/Defining-Our-Research-Methodology-DORy/3D-Microscopy-Metadata-Standards-3D-MMS.
